# Supplementary material for: Whole genome sequencing, characterization and analysis of coronene degrading bacterial strain Halomonas elongata
Source: PLoS One. 2025 Nov 19;20(11):e0334420. doi: 10.1371/journal.pone.0334420 (PMC12629441; doi:10.1371/journal.pone.0334420)
Supplement: S2 Fig — (DOCX) [file pone.0334420.s002.docx]

S2 Fig: Genes associated with superfamilies
