# Supplementary material for: Whole genome sequencing, characterization and analysis of coronene degrading bacterial strain Halomonas elongata
Source: PLoS One. 2025 Nov 19;20(11):e0334420. doi: 10.1371/journal.pone.0334420 (PMC12629441; doi:10.1371/journal.pone.0334420)
Supplement: S5 Fig — Where EC: 2.5.1.18 is glutathione S-transferase. (DOCX) [file pone.0334420.s005.docx]

*
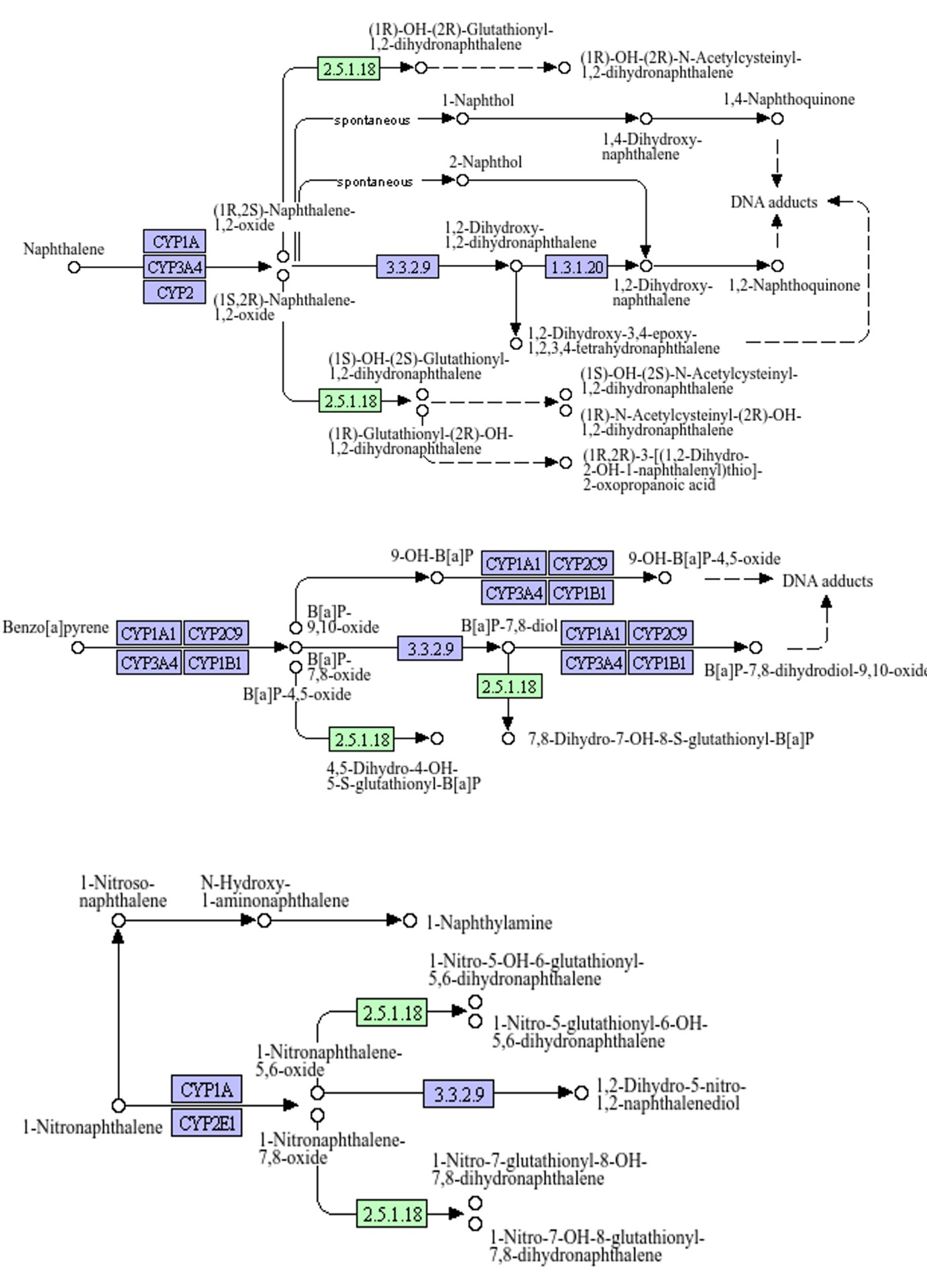
*

**S5 Fig: Pathway mapping of metabolism of xenobiotics by cytochrome P450 from KEGG-KASS** The green boxes indicate the genes that are present in our gene list while the blue boxes indicate those that are absent but should have been present
