# Supplementary material for: Whole genome sequencing, characterization and analysis of coronene degrading bacterial strain Halomonas elongata
Source: PLoS One. 2025 Nov 19;20(11):e0334420. doi: 10.1371/journal.pone.0334420 (PMC12629441; doi:10.1371/journal.pone.0334420)
Supplement: S1 Table — (DOCX) [file pone.0334420.s006.docx]

**S 1 Table Kraken2 taxonomical classification**

| **Percentage** | **Coverage** | **Assigned** | **Taxonomical Rank** | **NCBI ID** | **Scientific Names** |
| --- | --- | --- | --- | --- | --- |
| 13.21 | 1220 | 1220 | U | 0 | *unclassified* |
| 86.79 | 8018 | 1 | R | 1 | *root* |
| 86.75 | 8014 | 2 | R1 | 131567 | *cellular organisms* |
| 85.86 | 7932 | 13 | D | 2 | *Bacteria* |
| 85.51 | 7899 | 17 | P | 1224 | *Proteobacteria* |
| 84.82 | 7836 | 18 | C | 1236 | *Gammaproteobacteria* |
| 83.65 | 7728 | 2 | O | 135619 | *Oceanospirillales* |
| 83.57 | 7720 | 6 | F | 28256 | *Halomonadaceae* |
| 83.23 | 7689 | 39 | G | 2745 | *Halomonas* |
| 78.29 | 7232 | 0 | S | 2746 | *Halomonas elongata* |

Note: The first column is the percentage of the fragments that are rooted to the taxon, the second column is the number of fragments covered, the third column indicates the number of fragments that are directly assigned to the said taxon, the fourth column contains rank codes where S-species, G-Genus, F-Family, O-Order, C-Class, P-Phylum, D-Domain, R-Root, U-Unclassified, the fourth column consists NCBI taxonomic IDs and the last column has the Scientific Names.
