# Supplementary material for: Whole genome sequencing, characterization and analysis of coronene degrading bacterial strain Halomonas elongata
Source: PLoS One. 2025 Nov 19;20(11):e0334420. doi: 10.1371/journal.pone.0334420 (PMC12629441; doi:10.1371/journal.pone.0334420)
Supplement: S2 Table — (DOCX) [file pone.0334420.s007.docx]

**S 2 Table CRISPR sites**

| **Locus** | **Start** | **End** | **Length** | **orientation** | **Consensus Repeat sequence** |
| --- | --- | --- | --- | --- | --- |
| utg000001l_1 | 820200 | 820288 | 88 | Forward | TGATTACAAGTCAGCTGCTCTACCA |
| utg000005l_1 | 268455 | 268574 | 119 | Unknown | AGGACCAGTGCCCGGGTACCCCGGCCGGTG |
