# Supplementary material for: Whole genome sequencing, characterization and analysis of coronene degrading bacterial strain Halomonas elongata
Source: PLoS One. 2025 Nov 19;20(11):e0334420. doi: 10.1371/journal.pone.0334420 (PMC12629441; doi:10.1371/journal.pone.0334420)
Supplement: S3 Table — (DOCX) [file pone.0334420.s008.docx]

**S 3 Table RAST Annotation corresponding to aromatic compound metabolism**

| **Category** | **Subcategory** | **Subsystem** | **Role** |
| --- | --- | --- | --- |
| Metabolism of Aromatic Compounds | Peripheral pathways for catabolism of aromatic compounds | Biphenyl Degradation | biphenyl-2,3-diol 1,2-dioxygenase III-related protein |
|  |  | Benzoate degradation | Benzoate transport protein |
|  |  | Quinate degradation | 3-dehydroquinate dehydratase II (EC 4.2.1.10) |
|  | Metabolism of central aromatic intermediates | Catechol branch of beta-ketoadipate pathway | Muconate cycloisomerase (EC 5.5.1.1) |
|  |  |  | Succinyl-CoA:3-ketoacid-coenzyme A transferase subunit A (EC 2.8.3.5) |
|  |  |  | Succinyl-CoA:3-ketoacid-coenzyme A transferase subunit B (EC 2.8.3.5) |
|  |  | Salicylate and gentisate catabolism | Fumarylacetoacetase (EC 3.7.1.2) |
|  |  |  | Fumarylacetoacetate hydrolase family protein |
|  |  |  | Maleylacetoacetate isomerase (EC 5.2.1.2) |
|  |  | 4-Hydroxyphenylacetic acid catabolic pathway | 3,4-dihydroxyphenylacetate 2,3-dioxygenase (EC 1.13.11.15) |
|  |  |  | Homoprotocatechuate degradative operon repressor |
|  |  |  | 2-hydroxyhepta-2,4-diene-1,7-dioate isomerase (EC 5.3.3.-) |
|  |  |  | 5-carboxymethyl-2-hydroxymuconate semialdehyde dehydrogenase (EC 1.2.1.60) |
|  |  |  | Transcriptional activator of 4-hydroxyphenylacetate 3-monooxygenase operon, XylS/AraC family |
|  |  |  | 5-carboxymethyl-2-oxo-hex-3- ene-1,7-dioate decarboxylase (EC 4.1.1.68) |
|  |  |  | 2-oxo-hepta-3-ene-1,7-dioic acid hydratase (EC 4.2.-.-) |
|  |  |  | 5-carboxymethyl-2-hydroxymuconate delta-isomerase (EC 5.3.3.10) |
|  |  | Homogentisate pathway of aromatic compound degradation | Maleylacetoacetate isomerase (EC 5.2.1.2) |
|  |  |  | Homogentisate 1,2-dioxygenase (EC 1.13.11.5) |
|  |  |  | Fumarylacetoacetase (EC 3.7.1.2) |
|  |  |  | 4-hydroxyphenylpyruvate dioxygenase (EC 1.13.11.27) |
|  |  |  | Transcriptional regulator, IclR family |
|  |  | Central meta-cleavage pathway of aromatic compound degradation | 5-carboxymethyl-2-hydroxymuconate semialdehyde dehydrogenase (EC 1.2.1.60) |
|  |  |  | 3,4-dihydroxyphenylacetate 2,3-dioxygenase (EC 1.13.11.15) |
|  |  |  | 2-oxo-hepta-3-ene-1,7-dioic acid hydratase (EC 4.2.-.-) |
|  |  |  | 5-carboxymethyl-2-hydroxymuconate delta-isomerase (EC 5.3.3.10) |
|  | Metabolism of Aromatic Compounds - no subcategory | Aromatic Amin Catabolism | Phenylacetaldehyde dehydrogenase (EC 1.2.1.39) |
|  |  |  | 3,4-dihydroxyphenylacetate 2,3-dioxygenase (EC 1.13.11.15) |
|  |  | Gentisate degradation | Fumarylacetoacetate hydrolase family protein |
|  |  |  | Maleylacetoacetate isomerase (EC 5.2.1.2) |
