# Supplementary material for: Whole genome sequencing, characterization and analysis of coronene degrading bacterial strain Halomonas elongata
Source: PLoS One. 2025 Nov 19;20(11):e0334420. doi: 10.1371/journal.pone.0334420 (PMC12629441; doi:10.1371/journal.pone.0334420)
Supplement: Attachment — Submitted filename: Comments.pdf [file pone.0334420.s009.pdf]

General comments

1. Introduction needs some more expansion specially for the recent work published worldwide.
2. Where bacteria collected from? And full procedure regarding etc.
3. Background of the bacteria lacking from the manuscript.
4. Detailed procedure needed about whole genome sequencing and quality assessment.
5. Some headings/ subheading / paragraphs missing the major citation, please update accordingly.
6. There are several capitalizations between the sentence and heading. Authors should go throughout the manuscript for betterment of the article such as tile of fig 3, table 2 etc.
7. Discussion part needs more expansion especially relation with the recent work published.
8. References needs for formatting according the journal guidelines, there are several mistakes such as reference no 10 in the list. Fermentation 2022, **Vol** 8, 412 **Page** 260 2022;8:260.
9. Clarity of the figures blurry, not even able to understand the writeup materials.
