# Supplementary material for: Whole genome sequencing, characterization and analysis of coronene degrading bacterial strain Halomonas elongata
Source: PLoS One. 2025 Nov 19;20(11):e0334420. doi: 10.1371/journal.pone.0334420 (PMC12629441; doi:10.1371/journal.pone.0334420)
Supplement: Attachment — Submitted filename: RESPONSe_ReviewerPONE-D-25-12285.docx [file pone.0334420.s011.docx]

**RESPONSE TO THE REVIEWERS’ COMMENTs PONE-D-25-12285**

First of all,, we thank the reviewers for their valuable time in reviewing manuscripts. We have addressed their reviewer’s comment, and enclosed below is the point-by-point response to these comments.

# Reviewer #1

1. The abstract should provide a more concise yet informative summary, including key findings and their implications.

**Our response:**

*The abstract has been revised, and it is more concise and informative.*

2. The introduction needs a stronger rationale explaining why *Halomonas elongata* is particularly significant for coronene degradation compared to other bacteria. Key studies on bacterial PAH degradation should be included.

**Our response:**

*In light of the reviewer’s comments, we have expanded the introduction to incorporate new information on the degradation of PAHs in particular and that of the coronene in general. In addition, we listed around appropriate references that could help the readers to have a broader view of this topic.*

*Regarding the bacterial strain, it has been isolated as part of a different study,, and we have quoted this reference (Okeyode et al. 2023). However, in light of the reviewer comments (the same point has also been raised by reviewer 2), we have added the main information on the characteristics of this strain in the “Introduction section. All detailed information regarding this strain is summarised in Okeyode et al. 2003).*

3. More details on sequencing technology, genome assembly tools, and annotation methods would enhance reproducibility. Further, details on collection of sample and isolation of bacteria in in the beginning of materials and method are required. Name of the sequencing technology should be mentioned at line no 110 in full form

**Our response:**

- *We have addressed the comments related to Bioinformatics in the “Material & Method Section”.*
- *However, regarding the sources of samples and isolations of bacteria, as discussed in the previous section, this bacterial strain were isolated as part of a previous work, and the we have summarised the characteristics of this strain in the “Introduction”, and have quoted the reference linked to this work. We cannot add this information in the Material Methods since it is not part of the current work.*

4. The study aims to provide insights into the genetic mechanisms underlying coronene degradation. What molecular mechanism you unraveled for coronene degradation by genome sequencing of H elongata should be elaborated and may be presented using an appropriately designed flow chart.
5. **Our response:**

*Coronene is one of the most structurally complex PAHs, and currently, there is limited knowledge about the specific molecular mechanisms involved in its biodegradation. Initially, we hypothesized that the degradation of coronene might follow similar pathways to those well-characterized for other PAHs, such as naphthalene, phenanthrene, or benzo[a]pyrene. However, upon detailed genomic analysis of Halomonas elongata, no key enzymes typically involved in known PAH degradation pathways were found, from the annotated genome.*

*This unexpected result strongly suggests that H. elongata likely employs an alternative, previously undocumented pathway for coronene degradation. Identifying and confirming this novel route will require further experimental investigation, including metabolomic analyses to characterize intermediate degradation products. Only through such comprehensive metabolic profiling can we accurately delineate the complete biochemical pathway. These points have been highlighted in the last paragraph of the “Discussion Section.*

6. What is the basis of the cutoff 1e-06 in line 126?
7. **Our response:**

*The E-value cutoff of 1e-06 was chosen to ensure that only highly significant matches were retained during gene prediction. This stringent threshold minimizes false positives while maintaining sensitivity to detect true homologs, improving the accuracy and reliability of functional annotations.*

8. Line no. 154 k-value=1 should be justified.

**Our response:**

*A k-value of 1 was used in MobileOG-DB to ensure that even single, high-confidence matches to known mobile element proteins were detected, which is especially important for identifying potentially novel or incomplete mobile elements in environmental genomes.*

9. Table 1 should be properly placed for easy understanding, statistics related to Q20/Q30 should be given as minimum Q20 is an widely accepted Phred Score. The N50 value should also be provided in this table.

**Our response:**

- *Table location is based on the journal requirement. Thus, we wisht to stick to the journal instruction, unless the Editor states otherwise..*
- *Since this study used Oxford Nanopore long-read sequencing, traditional Phred-based Q20/Q30 scores—commonly reported in short-read technologies like Illumina—are not directly applicable. Nanopore sequencing evaluates read quality using different metrics (such as mean read quality scores, read length distributions, and base-calling accuracy), which are reported separately in our quality assessment.*

10. Table 2 is difficult to follow. Check the figures carefully. What is total length in Table 2. Sufficient information needs to be provided for each table and figures for making them self-explanatory.

**Our response:**

*We have carefully revised Table 2 to improve clarity, layout, and readability.*

- *The column “****Total length****” has now been clearly labeled and explained in the table legend. It refers to the cumulative length (in base pairs) of the sequences analysed for gene prediction by each respective tool.*
- *We have ensured that each tool’s name is clearly and consistently labeled (e.g., “PRODIGAL + EggNOG-mapper” and “GeneMarkS2 + EggNOG-mapper” presented on a single line).*
- *A comprehensive* ***table legend*** *has been added to explain each column, including:*

11. Most of the figures are of poor quality having very small fonts. The clarity of figures and tables should be ensured—some may need better labelling or explanations. Figure 5 may be represented as a 3d bar chart.
12. **Our response:**

- *All figures have been regenerated or replaced using high-resolution formats to ensure improved clarity in both digital and print formats.*
- *Fig 5 has been converted to a bar graph.*
- *To counter the resolution problem related to fig 7, it has been submitted in PNG*

13. Experimental validation of identified genes (e.g., knockout studies) would strengthen claims about metabolic pathways.
14. **Our response:**

*We fully agree that experimental validation—particularly through gene knockout studies—would greatly enhance the strength of our claims regarding the metabolic pathways involved in coronene degradation. However, we wish to clarify that this study was designed as a* ***computational genomic investigation*** *to identify potential genes and pathways implicated in high-molecular-weight PAH biodegradation using long-read whole genome sequencing and advanced bioinformatics tools. Importantly,* ***no known pathways for coronene degradation have been characterized to date****. Therefore, our current approach was guided by comparative genomic analysis, assuming similarity to other PAH-degradation mechanisms. Interestingly, genes traditionally associated with PAH degradation (e.g., dioxygenases involved in pyrene or benzo[a]pyrene metabolism) were* ***not found in our genome annotation****, suggesting the* ***existence of a novel or alternative degradation mechanism*** *in* Halomonas elongata*. This point have been highlighted in the final paragraph of “Discussion section”*

11. The discussion should include a more thorough comparison with previous studies on Halomonas elongata and other hydrocarbon-degrading bacteria. Functional analysis of specific genes responsible for coronene degradation should be elaborated. Metabolic pathways should be discussed, with a visual representation would improve comprehension.

**Our response:**

- *We have added a detailed comparison of our findings with previous research on other hydrocarbon-degrading Halomonas species, including H. organivorans, Halomonas sp. ML-15, and Halomonas sp. C2SS100. These comparisons highlight both the shared capabilities and the distinct genomic features of H. elongata 10SCRN4D, particularly with respect to its coronene degradation potential under hypersaline conditions.*
- *We elaborated on the annotation results by highlighting the presence of genes associated with xenobiotic metabolism and the absence of canonical PAH-degradation enzymes (e.g., aromatic ring-hydroxylating dioxygenases). We discussed the possible roles of monooxygenases, hydrolases, and dehydrogenases found in the genome, and proposed that H. elongata may employ an alternative or novel degradation route. The implications of these genes, along with the remaining 526 uncharacterized proteins, are discussed as promising targets for future experimental work.*
- *We agree that a visual representation would aid interpretation. However, due to the limited availability of coronene-specific degradation data and the incomplete pathway annotation obtained through KAAS, it was not possible to construct a complete predictive pathway. To address this, we have included* ***Supplementary Figure 5****, which displays the partial xenobiotic degradation pathway derived from KEGG annotations. This figure outlines the identified components of the strain’s potential metabolic capabilities and visually indicates the gaps and hypothetical nature of the degradation route.*
- *We acknowledged the limitation of relying solely on computational annotation and discussed the need for transcriptomic, proteomic, and metabolomic studies, as well as gene knockout and heterologous expression experiments. These points are now explicitly included the Discussion Section (in the final paragraph).*

12. The conclusion should emphasize the broader implications, such as potential applications in bioremediation and industrial use. Limitations and future research directions should be explicitly stated.

**Our response:**

*We have revised the conclusion section of our manuscript. Specifically, we have now highlighted the potential practical applications of Halomonas elongata in bioremediation of marine and hypersaline environments contaminated with polycyclic aromatic hydrocarbons; Discussed the prospective industrial applications of secondary metabolites; indicated the current lack of experimental validation; Recommended specific future studies involving targeted genetic experiments (such as gene knockout and overexpression) and comprehensive metabolite profiling.*

# Reviewer #2

1. Introduction needs some more expansion specially for the recent work published worldwide.

**Our response:**

*This comment is related to comment No 2 of the reviewer 1. We have expanded the Introduction section to incorporate more information on we have added relevant and appropriate references that the readers can you to gain insight on PAHs degradation. We have also added more information on the bacterial strain used in this study*

2. Where bacteria collected from?

**Our response:**

*As discussed earlier, this information has been added in the Introduciton.*

3. Background of the bacteria lacking from the manuscript.

**Our response:**

*This point, which as also raised by the Reviewer #1, has been addressed in the Introduction.*

4. Detailed procedure needed about whole genome sequencing and quality assessment.

**Our response:**

*This has been done (in Material & Methods).*

5. Some headings/ subheading / paragraphs missing the major citation, please update accordingly.

**Our response:**

*This has been checked and corrected*.

6. There are several capitalizations between the sentence and heading. Authors should go throughout the manuscript for betterment of the article such as tile of fig 3, table 2 etc.

**Our response:**

*We have followed the journal guidelines and the titles of figures and Tables have been crossed check and corrected.*

7. Discussion part needs more expansion especially relation with the recent work published.

**Our response:**

*This has been done. More discussion has been added.*

8. References needs for formatting according the journal guidelines, there are several mistakes such as reference no 10 in the list. Fermentation 2022, Vol 8, 412 Page 260 2022;8:260

**Our response:**

*This has been corrected now.*

9. Clarity of the figures blurry

**Our response:**

*The figures have been enhanced as soon as much we could, using available tools.*

END DOCUMENTS
