# Supplementary material for: Whole genome sequencing, characterization and analysis of coronene degrading bacterial strain Halomonas elongata
Source: PLoS One. 2025 Nov 19;20(11):e0334420. doi: 10.1371/journal.pone.0334420 (PMC12629441; doi:10.1371/journal.pone.0334420)
Supplement: Attachment — Submitted filename: Sept 9 Response Reviewer.docx [file pone.0334420.s012.docx]

# **Rebuttal Letter**

**Manuscript ID:** PONE-D-25-12285
**Title:** *Whole Genome Sequencing, Characterization, and Analysis of a Coronene-Degrading Bacterial Strain, Halomonas elongate*

We sincerely thank the reviewers for their time, effort, and constructive comments on our manuscript. We have taken note that Reviewer #2 found the revised version acceptable. Regarding Reviewer #1, some of his comments have already been discussed in our first revision, and in the rebuttal response below, we have carefully addressed all the concerns he has raised.

**Reviewer #1**

1. In Abstract: "This study extends that work through whole-genome sequencing utilizing Oxford Nanopore Sequencing, a long-read sequencing technology, followed by bioinformatics analysis to uncover the genetic mechanisms enabling this bacterium to degrade coronene effectively." I still don't find the uncovered genetic/molecular mechanism for coronene degradation by the bacterium.

**Our response:**

We thank the reviewer for this important observation. In the results section, we have clearly shown that our genomic analysis did not reveal a coronene degradation pathway. Instead, we identified genes associated with xenobiotic metabolism, suggesting that H. elongata may employ an alternative or novel degradation mechanism, and we have emphasized that this finding requires further experimental validation. To make this point clear, and in light of the reviewer's comment, we have summarised this information in this abstract.

2. Though authors have used the data from their previous research, in my opinion, still there should be a section on the sample collection where the process can be explained in brief by citing to their previous paper.

**Our response:**

“The same comment was raised during the first revision, and we addressed it by mentioning it in the Introduction. However, in light of the reviewer’s comment, we have now added this information in the Materials and Methods section under ‘DNA isolation, whole genome sequencing, and quality assessment.

3. "ONS technology" ONS should be expanded at its first instance.

**Our response:** This is done. It was explained in the Materials & Methods section, and has now been added to the abstract.

4. What is the basis of the cutoff 1e-06 in line 126?". My question was why it is 1e-06 not zero or any other value

**Our response**

*This comment was raised and discussed in the previous rebuttal. However, in light of the review comment, we have further clarified it (Material and Methods section). Zero is not a valid cutoff for BLAST; thus, an e-value of 1e-06 was chosen as a stringent threshold that minimizes false positives while retaining biologically meaningful homologs.*

5. Table 1 should be properly placed for easy understanding: statistics related to Q20/Q30 should be given as minimum Q20 is an widely accepted Phred Score. The N50 value should also be provided in this table." It's not about the location, it was about the rearrangement of the table in an easily readable way. Further, they have not presented the N50 value in the table.  In ONS many manuscripts presented the % of reads above Q=20. I understand the value will not be as much as Illumina. However, presenting these scores gives a better idea on read quality.

**Our response:**

*We thank the reviewer for this comment. Regarding Q20/Q30, as noted earlier, these metrics are Illumina-specific Phred quality scores and are not directly applicable to Oxford Nanopore sequencing data, which uses a different base-calling model and error distribution. Instead, ONT quality is evaluated through alternative measures (e.g., mean read quality, read length distributions, and base-calling accuracy), which we have already reported in Table 1. We have added q12 and q15 values.*

*Regarding the table readability, in preparing the previous revision, we organized Table 1 to present related parameters together (read quality, assembly statistics, and completeness) to allow straightforward interpretation. All critical information, including N50 and ONT-appropriate quality metrics, is provided in text, thus, we suggest to keep the current format.*

*As for the comment on NE50, the assembly N50 value has been included in Table 1.*

6. I still think experimental validation of identified genes would strengthen claims about metabolic pathways and thereby providing insights into the molecular mechanism.

**Our response:**

*The point raised by the reviewer is indeed relevant. However, as discussed in the manuscript, this study was designed as a computational genomic investigation using long-read sequencing and bioinformatics tools. We fully agree with the reviewer that experimental validation of the identified genes would strengthen our findings; however, such work was beyond the scope of the present study. As noted, further studies will be required to validate the proposed genes. We have clarified this point in the Discussion and Conclusion sections.*

**Reviewer #2**

We thank Reviewer #2 for their supportive feedback and note that no further comments were raised.
